# Supplementary material for: Campylobacter jejuni and Campylobacter coli infection, determinants and antimicrobial resistance patterns among under-five children with diarrhea in Amhara National Regional State, Northwest Ethiopia
Source: PLoS One. 2024 Jul 3;19(7):e0304409. doi: 10.1371/journal.pone.0304409 (PMC11221748; doi:10.1371/journal.pone.0304409)
Supplement: Attachment — Submitted filename: Response to Reviewers.docx [file pone.0304409.s001.docx]

**Response to Reviewers**

To academic editor

We appreciate the assessment carried out by editorial team. We reviewed and made necessary correction as follows

1. Please ensure that your manuscript meets PLOS ONE's style requirements, including those for file naming.

**Response:** Thank you! we reviewed it again and amended as per PLOS ONE's style requirements

2. Please note that funding information should not appear in any section or other areas of your manuscript. We will only publish funding information present in the Funding Statement section of the online submission form. Please remove any funding-related text from the manuscript.

**Response:** Thank you! We removed funding related text from the manuscript

3. Thank you for stating the following financial disclosure:
      "University of Gondar, Animal Health Institute (AHI), and International Livestock Research Institute (ILRI) supports for data collection and laboratory investigations."

Please state what role the funders took in the study.

**Response:** Thank you! We amended the financial disclosure as **“**University of Gondar, Animal Health Institute (AHI), and International Livestock Research Institute (ILRI) supports for data collection and laboratory investigations. The funders had no role in study design, data collection and analysis, decision to publish, or preparation of the manuscript”.

4. We note that your Data Availability Statement is currently as follows: All relevant data are within the manuscript and its Supporting Information files

**Response:** Thank you! Sorry for encountered mistake. We have no supporting files and we stated it as “All relevant data are within the manuscript”

5. PLOS requires an ORCID iD for the corresponding author in Editorial Manager on papers submitted after December 6th, 2016. Please ensure that you have an ORCID iD and that it is validated in Editorial Manager. To do this, go to ‘Update my Information’ (in the upper left-hand corner of the main menu), and click on the Fetch/Validate link next to the ORCID field. This will take you to the ORCID site and allow you to create a new iD or authenticate a pre-existing iD in Editorial Manager. Please see the following video for instructions on linking an ORCID iD to your Editorial Manager account: <https://www.youtube.com/watch?v=_xcclfuvtxQ>
6. Your ethics statement should only appear in the Methods section of your manuscript. If your ethics statement is written in any section besides the Methods, please delete it from any other section.

**Response:** Thank you! We removed ethics statement from other section and stated only in method section.

7. Please ensure that you refer to Figure 1 in your text as, if accepted, production will need this reference to link the reader to the figure.

**Response:** Thank you! We have already stated in Line No 273

8. Please include a caption for figure 1.

**Response:** Thank you! We have already stated in Line No 277. But changed the word ‘picture’ to ‘figure’

9. We note you have included a table to which you do not refer in the text of your manuscript. Please ensure that you refer to Table 6 in your text; if accepted, production will need this reference to link the reader to the Table.

**Response:** Thank you! We corrected the order of each table and referred accordingly

10. Please review your reference list to ensure that it is complete and correct. If you have cited papers that have been retracted, please include the rationale for doing so in the manuscript text, or remove these references and replace them with relevant current references. Any changes to the reference list should be mentioned in the rebuttal letter that accompanies your revised manuscript. If you need to cite a retracted article, indicate the article’s retracted status in the References list and also include a citation and full reference for the retraction notice.

**Response:** Thank you! We carefully reviewed and all listed references are complete and cited. We had no retracted papers.

**Reviewer #1**

We would like to express our gratitude to reviewer 1 for providing the important comments on the issue of confidence interval

1. Line 45: Among the Campylobacter isolates, C. jejuni were commonly isolate (73.3%).......
   Kindly add 95%CI to all proportions in the manuscript either in the text or the tables. This provides a better expression of the proportion uncertainty.

**Response:** Thank you so much and we appreciate the concern! We calculated Confidence interval (CI) and added in both text and tables which indicated with yellow color highlight, but 73.3% and 26.7% were calculated to show the frequency of specious among 30 isolates.

**Reviewer #2**

We express our gratitude to reviewer 2 for providing a thorough overview of our research and highlighting both its merits and drawbacks. We are committed to improving our manuscript by implementing the required revisions. We have taken the time to provide a detailed response that addresses each raised concern.

1. **Language** - there are numerous grammatical errors in the manuscript that are distracting from the important message. The authors could seek professional language editing services to improve the quality of this manuscript.

**Response**: Thank you! We have identified the problem and made corrections at both the word and paragraph levels. Furthermore, we have taken punctuation into account. The highlighted portions are in yellow for your review.

2. The **abstract** methods do not include some crucial information about how the study was conducted and only focusses on laboratory methods.

**Response:** Thank you! We included the missed information methods of the abstract and highlighted with yellow color. The number of the word for abstract limited us. (37-39)

3. **Introduction** - although the authors mention that they used an advanced laboratory technique (MALDI-TOF MS) to identify the organisms, they do not mention what is the advantage of this method over traditional methods.

**Response:** Thank you! We included about advantage of the MALDI-TOF (Line no 104-106)

4. **Methods** - source population - the two sentences in this section are very similar and repetitive.

**Response:** Thank you and accepted and **w**e now clearly stated to avoid similarity (Line 128-130)

5. **Sampling technique** - it is not very clear how the children were selected and what case definitions of diarrhoea were applied.

**Response:** Thank you! We restated and kindly refer to Line No 137-142

**Data analysis** - the cut off for including variable in a multivariate model is given as 0.25. This threshold is very high; the authors should consider repeating this analysis using a lower threshold.

**Response:** Thank you and we accepted and reviewed. After conducting a bivariate logistic regression analysis, we recalculated our candidate variable and were pleased to find that the results remained unchanged, with all variables falling below 0.2. This led us to replace 0.25 with 0.2.

**Results -** There are two Table 4s in this section. One appears after Table 6. The abbreviations used in the second Table 4 should be spelt out in a footnote below the table.

**Response:** Thank you and accepted and corrected accordingly

6. **Discussion** - the authors should revise the first paragraph in this section to summarise the main findings in the study.

**Response:** Thank you! We revised the first paragraph accordingly, Line No 326-333

The authors note that the prevalence of campylobacter spp was similar to that found in other studies in Ethiopia but higher than other countries such as Kenya. Could this be attributed to the different laboratory techniques? In general, this section focusses so much on comparing study findings with previous study at the expense of explaining the implications of their findings for public health.

**Response:** Thank you and we appreciate your constructive comment. There are various potential factors that may contribute to the disparity between our findings and previous studies. Among these factors, the laboratory method could be one of the probable reasons due to its specificity and sensitivity. The utilization of state-of-the-art technology can significantly impact the prevalence of campylobacters. Additionally, we have also mentioned other potential factors that could account for the variation the prevalence of Campylobacter infections. (L341-344)

**Conclusions** - the following statement is not fully back up by data presented in this manuscript:
"Culture and sensitivity test should be carried out prior to medication of diarrheal patients"

**Response:** Thank you and we accept the commend and corrected accordingly (L416-424)
