# Supplementary material for: Campylobacter jejuni and Campylobacter coli infection, determinants and antimicrobial resistance patterns among under-five children with diarrhea in Amhara National Regional State, Northwest Ethiopia
Source: PLoS One. 2024 Jul 3;19(7):e0304409. doi: 10.1371/journal.pone.0304409 (PMC11221748; doi:10.1371/journal.pone.0304409)
Supplement: Attachment — Submitted filename: Campyello bacter.docx [file pone.0304409.s002.docx]

**Title:** Campylobacter jejuni and Campylobacter coli infection, determinants and antimicrobial resistance pattern among under-five children with diarrhea in Amhara National Regional State, Northwest Ethiopia

The title is very important and it is tries to address the current hot topics and the study makes significant contributions to the study area. But it needs some modifications like

Campylobacter jejuni and coli infection, determinants and antimicrobial resistance pattern among under-five children with diarrhea in Amhara National Regional State, Northwest Ethiopia

Campylobacter species infection, determinants and antimicrobial resistance pattern among under-five children with diarrhea in Amhara National Regional State, Northwest Ethiopia

- Check affiliation of each Author and the corresponding number.

**Abstract**

- **The whole manuscript needs language revision from example in the abstract section the first sentence is fragmented should be revised and the term exploration is more of for qualitative study (line 29-32).**
- **In the abstract section there is repeation of the objective both in the background section and the objective section please make or write one section.**

**Methods:**

- **The study sites were selected using a random sampling technique, while the study subjects were included using a convenient sampling technique. Data** were **collected using a structured questionnaire section needs revision.**

**Result:**

- The prevalence of C. jejuni and C. coli among under-five children was 5.1% (CI: 3.0-7.0%) and 1.9% (CI: 0.7-3.3%) (line #45) add respectively avoid fragmentation of the sentence.
- The resident avoid the (line #45-46)
- One-third of the Campylobacter isolates (33.3%) (Line # 47-48) use consistent expressions of the frequencies either number in words and percentage or number in a numerical value and percentage.

**Conclusion:**

- The prevalence of Campylobacter species was relatively low relative to what. However, a high rate of ciprofloxacin and tetracycline-resistance strains was identified relative to what is your reference. Continuous surveillance on antimicrobial resistance and health education personal and environmental hygiene should be implemented in the community. Indicate the responsible bodies and future perspective for the researchers.

**Introduction**

- **In this section please you’re referencing for indicating burden of diarrheal cases in sub-Saharan Africa and Asia you use a single reference is that to mean that 90% of prevalence of diarrhea**
- **Paragraph two line # 69 give the apperoperate place for and**
- **Chickens, due to their affordability, are widely accessible in communities then what please clarify for the reader such fragmented sentence (line #73)**
- **There are a lot of studies conducted on the prevalence of and drug resistance pattern of this bacterium in Ethiopia. What makes your study different from this studies**
- **The rationale of the problem and its determinants in Ethiopia particularly in the study area is not stated in the introduction part, hence needs revision.**

**Methods**

**Source and study population**

**The source population was all children under-five years of age. The study population was all children under-five with diarrhea attending randomly selected government health centers**

**Sample size determination**

- **The sample size was determined by applying single population formula, n = Z^2^ p (1-p)/ d2. with consideration of design effect by taking a prevalence of 15.4% from previous study in Gondar among under five diarrheic patients [19] at 95% confidence interval and precision of 5%. Suggestion you can rewrite in this way to avoid repeation of words and to clarify from where you take your prevalence.**

**Sampling technique**

- You selected Kebeles from urban and Peri-urban areas of these two sites how many of the Kebeles were from urban and per-urban and the distribution of selected children in both of the Kebeles because the exposure rate or risk factors. Include it please **(Line #136-142).**
- The recruitment of under-five children with diarrhea was done using a convenient sampling technique, which involved selecting how do you see the calculated sample size and convenient sampling.

**Transportation and processing of the specimen**

- Approximate temperature of 4oC is it **40°C or 4°C** Correct it Line # 153

**Results**

Socio-demographic characteristics of under-five children

- **Table: 1,2** Socio-demographic characteristics of the under five children and their parents/guardians in the Amhara National Region state, Northwestern Ethiopia, November, 2022 to April, 2023. **Please follow the Journal policy for wording for the title.**

**Prevalence of Campylobacter species among fewer than five children**

- The prevalence of C. jejuni and C. coli among under-five children was 5.1% (CI: 3.0-249 7.0%) and 1.9% (CI: 0.7-3.3%). Of the Campylobacter species, C. jejuni were more frequent (73.3 %) than C. coli (26.7%). Check for the statistics and make it clear for reader.
- The prevalence of Campylobacter species among male and female participants was nearly similar (P=0.81). What does it mean?
- For me Table 4 is not required because all of the ideas or results are mentioned in the text so need of repeation.
- **Table 6:** Shorten the title and Index the sort terms in the table.

**Discussion**

- Please check language, fragmented sentences, punctions etc

**Conclusion**

- The conclusion should be based on the findings of the study, recommendations for responsible bodies, and future research prospective for researchers and readers so it needs revision.

**Author’s contribution** Check the Journal author contribution criteria

**References:** Check the journal referencing style, punctuation and etc..
